# DEAD-Box Helicase 4 (Ddx4)^+^ Stem Cells Sustain Tumor Progression in Non-Serous Ovarian Cancers

**DOI:** 10.3390/ijms21176096

**Published:** 2020-08-24

**Authors:** Stella D’Oronzo, Erica Silvestris, Domenica Lovero, Paola Cafforio, Loren Duda, Gennaro Cormio, Angelo Paradiso, Raffaele Palmirotta, Franco Silvestris

**Affiliations:** 1 Department of Biomedical Sciences and Human Oncology–Section of Internal Medicine and Clinical Oncology, University of Bari Aldo Moro, 70121 Bari, Italy; domenica.lovero@uniba.it (D.L.); paola.cafforio@uniba.it (P.C.); raffaelepalmirotta@gmail.com (R.P.); 2 I.R.C.C.S-Giovanni Paolo II Cancer Institute, 70124 Bari, Italy; ericasilvestris85@gmail.com (E.S.); gennaro.cormio@uniba.it (G.C.); a.paradiso@oncologico.bari.it (A.P.); 3 Department of Pathology, University of Foggia, 71122 Foggia, Italy; lorenduda@live.it; 4 Department of Biomedical Sciences and Human Oncology–Section of 2nd Unit of Obstetrics and Gynecology, University of Bari Aldo Moro, 70121 Bari, Italy

**Keywords:** Ddx4, follicle-stimulating hormone, non-serous epithelial ovarian cancer, gene expression, next-generation sequencing

## Abstract

DEAD-Box Helicase 4 (Ddx4)^+^ ovarian stem cells are able to differentiate into several cell types under appropriate stimuli. Ddx4 expression has been correlated with poor prognosis of serous ovarian cancer (OC), while the potential role of Ddx4^+^ cells in non-serous epithelial OC (NS-EOC) is almost unexplored. The aim of this study was to demonstrate the presence of Ddx4^+^ cells in NS-EOC and investigate the effect of follicle-stimulating hormone (FSH) on this population. Increased Ddx4 expression was demonstrated in samples from patients with advanced NS-EOC, compared to those with early-stage disease. Under FSH stimulation, OC-derived Ddx4^+^ cells differentiated into mesenchymal-like (ML) cells, able to deregulate genes involved in cell migration, invasiveness, stemness and chemoresistance in A2780 OC cells. This effect was primarily induced by ML-cells deriving from advanced NS-EOC, suggesting that a tumor-conditioned germ cell niche inhabits its microenvironment and is able to modulate, in a paracrine manner, tumor cell behavior through transcriptome modulation.

## 1. Introduction

Ddx4 is a member of the DEAD box protein family physiologically involved in the gametogenesis, division and development of primordial germ cells, as well as cellular growth [1]. This protein has also been associated with brain tumor formation in Drosophila [2] and tumor progression of human epithelial ovarian cancers (OCs), with its increased expression detectable in high-grade serous tumors (HGSOC) being correlated with poor prognosis [3].

As an oogonial germline marker, Ddx4 is expressed by ovarian stem cells (OSCs) as membrane protein, mainly in early stages, and only at the cytoplasmic level after their in vitro differentiation to oocyte-like cells [4,5,6]. OSCs are spherical cells slightly larger than the very small embryonic-like stem cells (VSELs) [7], and their stemness is supported by the expression of typical markers as OCT4, NANOG, SSEA1 and SSEA4 [8] as well as by others also shared by cancer stem cells, including CD44, LGR5, and CD133 molecules [8,9].

OSCs are reported to home within the ovarian cortex even in post-menopausal women and are suspected to support neo-oogenesis and primordial follicle assembly in response to the follicle stimulating hormone (FSH) through its alternatively spliced receptor variant 3 (FSHR3) [10]. These cells have been demonstrated to differentiate in vitro to large oocyte-like cells resembling mature oocytes [11] and thus to be suitable to restore fertility in pharmacologically sterilized animal models [12]. On the other hand, OSCs are suspected to contribute to epithelial OC development in relation to their high sensitivity to FSH [13,14].

FSH is considered a major hormone involved in OC pathogenesis in relation to its physiological serum elevations in menopause and for the higher serum levels detectable in OC patients with poor prognosis [15,16,17]. Furthermore, FSH has been described to support the growth of OC metastasis by reinforcing neo-angiogenesis through the autologous secretion of vascular endothelial growth factor (VEGF), by affecting several oncogenic pathways that regulate both proliferation and invasion, and by suppressing apoptosis in cancer cells [18]. The role of FSH in ovarian carcinogenesis has further been demonstrated by the delayed tumor progression of experimental OC in mice, obtained by a DNA vaccine breaking the immune tolerance to FSHR [19].

However, the contribution of FSH in OC progression also includes its putative capacity to induce the epithelial to mesenchymal transition (EMT) in epithelial OC cells [20,21,22]. Achievement of a motile and invasive phenotype by these tumor cells is supported by the abundant FSH bioavailability in post-menopausal age, which deregulates the E-cadherin expression as well as the molecular signaling driven by transforming growth factor β (TGF-β), Wnt/β-catenin, NOTCH, and PI3K/Akt pathways, resulting in typical cancer events, such as the disruption of epithelial integrity, loss of cell–cell junctions and, ultimately, the dissemination of tumor cells in blood [22]. Furthermore, FSH primes a proliferative effect not only in normal ovarian surface epithelial cells but also in malignant cells, thus supporting its crucial role in ovarian tumorigenesis in line with the gonadotropin hypothesis [23]. In this regard, it has also been reported that FSH and luteinizing hormone (LH) promptly activate the PI3K/Akt pathway to promote EMT and induce OC cell invasiveness [24] and that this effect can be abolished by a selective PI3K inhibitor [22].

Functional studies exploring the hormone sensitivity of CD133^+^/Ddx4^+^VSELs showed that, in response to FSH, such a population of OSCs undergoes active proliferation resulting in formation of large tumor-like clusters [14]. Moreover, under other stimuli, VSELs also differentiate to adipocytes, osteoblasts or neural cells, as an effect of their plasticity and pluripotency [14]. To this, it has been described that the Ddx4^+^ population embraces a mesodermal commitment typical of mesenchymal stem cells (MSCs), since they phenotypically express CD44, CD73, CD90, CD105 and CD146, while under specific stimuli may also differentiate into cells belonging to other germ layers [25]. Hence, in relation to such a suggested native proclivity toward a mesenchymal evolution, it is conceivable that the Ddx4^+^ cell enrichment in OC is related to the FSH-primed differentiation of OSCs to intra-tumor stromal cells, to sustain OC growth and expansion.

The present study was thus aimed at investigating the role of Ddx4^+^ OSCs in post-menopausal women with regard to the ovarian carcinogenesis, particularly in non-serous epithelial ovarian cancer (NS-EOC). We had the possibility to investigate these cells in both not tumor ovaries and in groups of differently staged NS-EOCs, and found their enrichment particularly within the stromal component of aggressive tumors. Thus, we explored the hypothesis that, in response to FSH stimulation, Ddx4^+^ OSCs differentiate to mesenchymal-like (ML) cells capable to regulate the tumor cell growth and aggressiveness by inflecting their transcriptome.

## 2. Results

### 2.1. Patients’ Distribution

Table 1 includes major clinical data from both cohorts, namely controls and OC patients. As shown, control patients underwent surgery for benign gynecological diseases, whereas those subgrouped in A1 and A2 were diagnosed with NS-EOCs, divided in relation to their International Federation of Gynecology and Obstetrics (FIGO) clinical stage and tumor histotypes as “clear cell”, “mucinous” and “endometrial” carcinoma (clinical-pathological details are listed in Supplementary Table S1).

### 2.2. Ddx4 is Highly Expressed in A2780 Cells and OC Tissue

**Figure 1:**
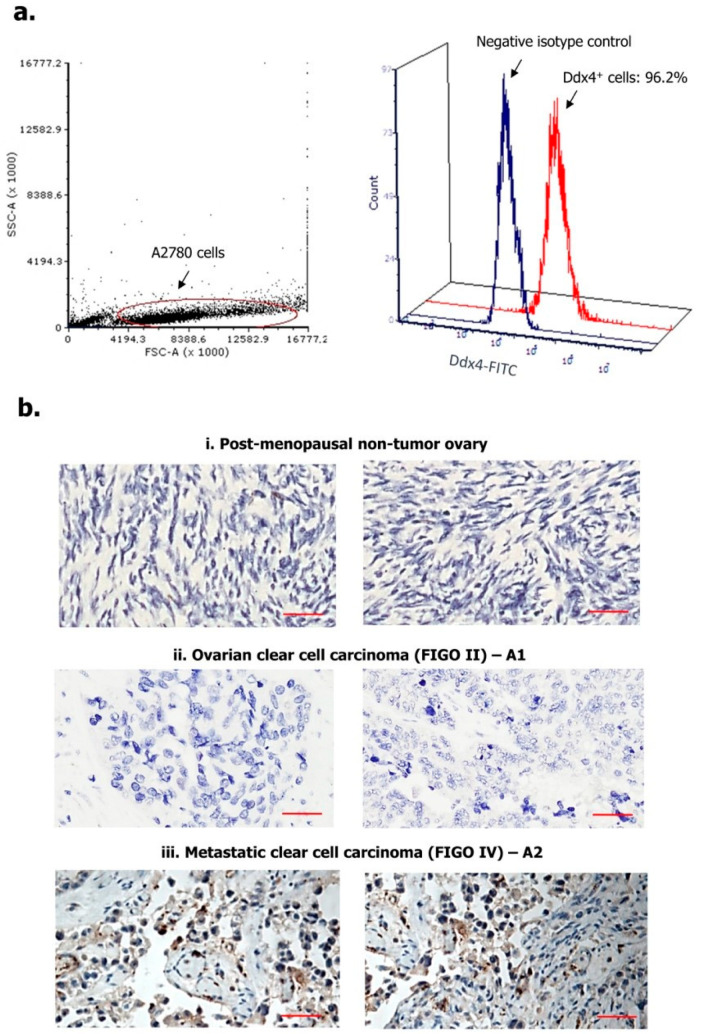
Expression of Ddx4 in A2780 cells (**a**) and ovarian samples (**b**). As a model of non-serous epithelial ovarian cancer (NS-EOC), A2780 cells were investigated by flow-cytometry, for the expression of Ddx4, after fixation and permeabilization (**a**). As shown, up to 96.2% of cells inside the gated region exhibited Ddx4 expression (Fluorescein Isothyocianate (FITC) signal). The antigen was also detected by Immunohistochemistry (IHC) in both healthy ovaries derived from post-menopausal women (**i**) and NS-EOC samples (**ii**, **iii**). Compared to the almost negative control samples in (**i**), representative panels from A1 (**ii**) and A2 (**iii**) patients exhibit higher Ddx4 expression, both in stromal and in cancer cells at the cytoplasmic level (**b**). In particular, the strongest staining intensity for Ddx4 was found in samples from patients included in the A2 group (International Federation of Gynecology and Obstetrics (FIGO) IV stage, *n* = 2), while moderate (++) to weak (+) scores occurred in A1 (FIGO I-II, *n* = 2). Scale bars = 50 µm.

We first assessed Ddx4 expression in A2780 cell line, as a model of NS-EOC, and in patients’ OC specimens. As shown in Figure 1a, following cell fixation and permeabilization, Ddx4 was revealed by flow cytometry at high cytoplasmic levels in almost the entire A2780 cell population (96.2%), while a variable expression of the antigen was found by immunohistochemistry (IHC) in patient-derived tumor samples (Figure 1b). Indeed, according to the mean percentage of Ddx4^+^ cells, ovarian specimens from the control group were found apparently negative or very weakly positive (Figure 1(bi): representative panels from two control pts), whereas a higher, even if variable, degree of Ddx4 expression was recorded between A1 and A2 groups (Figure 1(bii,biii)). In fact, in patients with early-stage OC (A1 subgroup), we found an average of 17.4 ± 4.1% Ddx4^+^ cells in contrast with 61.6 ± 12.5% detected in OC samples from patients with advanced disease (A2). Furthermore, besides such a discrepancy, we also observed a differential staining intensity of Ddx4 by IHC. In fact, while in samples from the A1 group a weak intensity (score +) occurred, the majority (8/14) of those from A2 were classified with the highest score (+++) (Table 1).

Figure 1b-ii and iii show representative patterns of two samples from A1 (pts n. 2 and 3) and A2 (pts n. 10 and 15) subgroups, respectively, depicting the differential scores. Notably, Ddx4 staining was predominantly cytoplasmic, though the perinuclear localization was also observed. However, a high signal intensity of Ddx4 occurred also in stromal cells within the tumor microenvironment, in particular in several samples of invasive OCs grouped in A2.

This first set of experiments suggested that Ddx4 was largely expressed by advanced NS-EOCs, both as percentage of positive cells and staining intensity, while modestly occurring in OC specimens from patients with minimally invasive and locoregional disease.

### 2.3. OC Samples Include Variable Amounts of Ddx4^+^ Cells

By applying a previously-described protocol [11], we isolated Ddx4^+^ cells from fresh ovarian samples of similar size of approximately 1.2 cm^3^ and, in line with IHC results, differential values were obtained between the two groups of OC patients. The mean number of Ddx4^+^ cells isolated from OC fragments belonging to the A1 group was 2.01 ± 0.9 × 10^5^ cells, whereas it was significantly higher in samples from A2 patients (5.06 ± 0.7 × 10^5^ cells) according to Student’s t test (*p* < 0.05).

**Figure 2:**
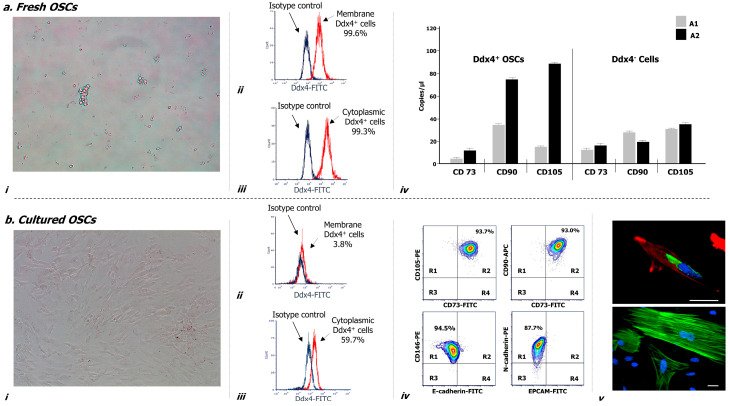
Morphological and molecular characterization of Ddx4^+^ cells derived from NS-EOC samples, before (**a**) and after two weeks of culture, in the presence of follicle-stimulating hormone (FSH) and epidermal growth factor (EGF) (**b**). (**a**) After their isolation from NS-EOC samples, Ddx4^+^ cells appeared small and round, singularly distributed or forming small aggregates (**a-i**). Moreover, the majority of these cells (>90%) expressed Ddx4, at both membrane (**a-ii**) and cytoplasmic levels (**a-iii**); this was evaluated by flow cytometry either before (**a-ii**) or after permeabilization (**a-iii**) of isolated Ddx4^+^ cells, processed with an FITC-conjugated anti-rabbit antibody (in red: positive staining for Ddx4; in blue: isotype control). The native tendency of Ddx4^+^ cells to undergo ML differentiation was revealed by droplet digital PCR (ddPCR), which showed the baseline expression of CD73, CD90, and CD105 genes in Ddx4^+^ cells from OC patients, at a significantly higher extent (*p* < 0.02) in those derived from A2 tumors. On the other hand, Ddx4-negative cells from both groups of OC patients expressed significantly lower levels of the mesenchymal markers than those found in A2-derived Ddx4^+^ cells (*p* < 0.02) (**a-iv**). The results are expressed as mean values ± standard deviation (SD) of experiments performed in triplicate. (**b**) After the first week of culture, in the presence of FSH and EGF, tumor-derived Ddx4^+^ cells acquired a fibroblast-like shape (**b-i**), while varying their Ddx4 expression, which decreased on the cell membrane (**b-ii**) but was maintained in the cytoplasm of 59.7% cells (**b-iii**). Flow-cytometry analysis revealed the concomitant expression of multiple mesenchymal markers on the majority of 14 day-cultured ML-Ddx4^+^ cells, whereas the expression of either E-cadherin or Epithelial cell adhesion molecule (EPCAM) was poorly detectable. The representative contour plots in (**b-iv**) show in R2 quadrants of the upper panels the co-expression of CD73 with CD105 (93.7%), and CD73 with CD90 (93.0%), in the analyzed cell population. Moreover, as shown in the lower panels, related to cells double-stained for mesenchymal and epithelial markers, the localization of the cell population in the R1 quadrant suggested that only CD146 and N-cadherin were expressed, whereas E-Cadherin and EPCAM were detected on a very small cell fraction (range: 1–5%) (**b-iv**). We further confirmed the acquisition of a fibroblast-like shape by confocal microscopy (**b**-**v**), together with cytoplasmic and perinuclear Ddx4 localization (up: Ddx4 in green, actin in red, nuclei in blue; down: actin in green, nuclei in blue). Scale bars: 20 µm.

Figure 2 illustrates morphological and phenotypical features of Ddx4^+^ cells, both before (a) and after culture in vitro (b). As depicted, after their isolation these cells appeared small, round, translucent and variably distributed as single cells or in small aggregates (a-i), and were almost all (>99%) expressing Ddx4, at both membrane (a-ii) and cytoplasmic levels (a-iii).

Furthermore, droplet digital PCR (ddPCR) analysis evaluating the mesenchymal commitment (a-iv) revealed that Ddx4^+^ cells from the A2 group expressed significantly higher levels of three mesenchymal markers (i.e., CD73, CD90 and CD105) compared to those isolated from samples grouped in A1 (*p* < 0.02). Moreover, Ddx4-negative populations from both groups of OC patients expressed significantly lower levels of the mesenchymal markers than those found in A2-derived Ddx4^+^ cells (*p* < 0.02) (a-iv).

This analysis provided evidence that intra-tumor Ddx4^+^ cells were already activated in their commitment to differentiate toward the mesenchymal lineage, especially when derived from the microenvironment of an advanced NS-EOC, compared to not metastatic OCs from the A1 subgroup.

### 2.4. Cultured Ddx4^+^ Cells from NS-EOCs Show Morphologic and Phenotype Patterns of Mesenchymal Cells

Cultures of Ddx4^+^ cells from OC samples were maintained for up to 14 days in the presence of FSH and Epidermal Growth Factor (EGF). After the first week, these cells acquired a typical elongated fibroblast-like shape (Figure 2(b-i)) and underwent active proliferation until reaching up to 90% of confluence. We also observed variations in their Ddx4 expression and phenotype. In fact, the membrane expression of Ddx4 fell down to less than 4% (b-ii), whereas, although at lower magnitude than in non-cultured cells, the molecule was consistently maintained in the cytoplasm of approximately 60% cultured cells, as depicted in the representative sample in Figure 2(b-iii).

With the purpose to better characterize these cells, we analyzed by flow cytometry the expression of mesenchymal markers, which was found high for CD73 (93.5 ± 2.1%), CD90 (97.0 ± 0.8%), CD105 (96.1 ± 1.2%), CD146 (98.9 ± 0.1%) and N-cadherin (89.2 ± 0.7%), whereas the expression of either E-cadherin or Epithelial cell adhesion molecule (EPCAM) was lower (4.4 ± 0.5% and 1.5 ± 0.7%, respectively). Moreover, flow cytometry analysis revealed the concomitant expression of multiple mesenchymal markers on the majority of 14 day-cultured Ddx4^+^ cells, whereas the expression of either E-cadherin or EPCAM was poorly detectable. The representative contour plots in Figure 2(b-iv), as two-dimensional flow cytometry pictures for multi-stained samples, show in R2 quadrants of the upper panels the concomitant expression of CD73 with CD105, and CD73 with CD90 on the same cell population, stained with the relative antibody mixture. In particular, approximately 94% of these cells exhibited a mesenchymal phenotype. Moreover, as shown in the lower panels, depicting cells double-stained for both mesenchymal and epithelial markers, the localization of the cell population in R1 quadrant suggested that only CD146 and N-cadherin were expressed, whereas E-Cadherin and EPCAM were detected on a very small residual cell fraction (range: 1–5%).

We further confirmed the acquisition of a fibroblast-like shape by cultured Ddx4^+^ cells by confocal microscopy (b-v), which showed their spindle-like morphology with enriched cytoplasmic and perinuclear Ddx4 localization (up: green staining), together with a typical F-actin distribution along the stretched cytoskeleton (up: red staining; down: green staining).

These data further supported the observation that Ddx4^+^ cells derived from the NS-EOC microenvironment were committed to mesenchymal evolution and, under stimulation with FSH and EGF, exhibited phenotypical and morphological mesenchymal patterns.

### 2.5. ML-Ddx4^+^Cell Effect on OC Cell Proliferation

**Figure 3:**
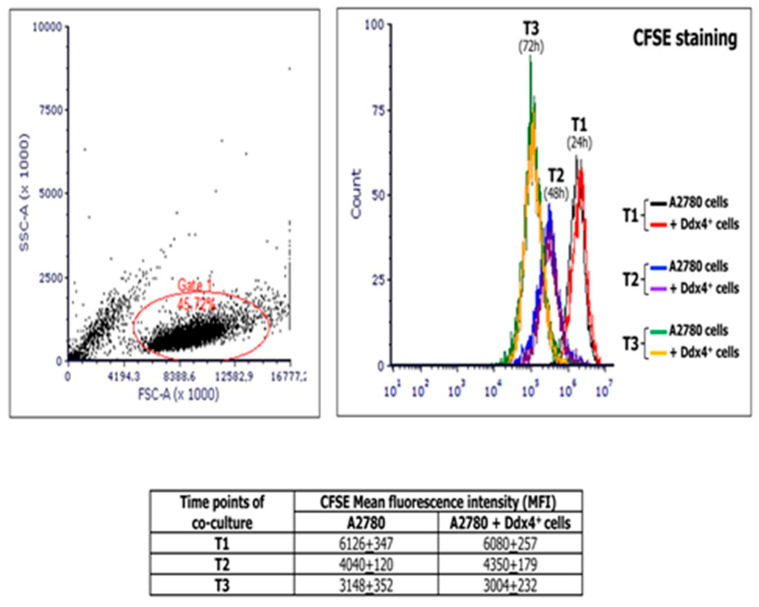
Measurement of the A2780 proliferation extent at different time points of co-culture with Ddx4^+^ cells derived from a representative A2 patient. The image shows carboxyfluoresceinsuccinimidyl ester (CFSE) assay results, in terms of mean fluorescence intensity (MFI), derived from co-culture experiments performed in triplicate by using ML-Ddx4^+^ cells isolated from a representative A2-OC patient (pt #12). As depicted, after cell cycle synchronization, A2780 cells exhibited an homogeneous doubling time of 24 h and hystograms of co-cultured cells and controls were perfectly overlapping at each time point (numbers in the table correspond to MFI values).

MSCs are generally assumed to support the tumor cell proliferation [26,27]. Thus, to investigate the modulation of tumor cell viability and proliferation by ML cells, we co-cultured A2780 population with ML-Ddx4^+^ cells derived from either healthy ovaries or NS-EOCs, separated by pored inserts and, at different time points of the culture, namely 24 (T1), 48 (T2), and 72 (T3) h, we measured the extent of cell divisions in A2780 cells by the carboxyfluoresceinsuccinimidyl ester (CFSE) assay. Figure 3 shows the results of mean fluorescence intensity (MFI) values derived from experiments performed in triplicate using ML-Ddx4^+^ cells isolated from a representative A2-OC patient, showing that, after cell cycle synchronization, A2780 cells exhibited a homogeneous doubling time of 24 h. However, hystograms of co-cultured cells and controls were perfectly overlapping at each time point, thus demonstrating that ML-Ddx4^+^ cells preserved OC cell viability and proliferation, although the latter was not increased, compared to control values. Similar results were obtained with ML-Ddx4^+^ cells from control patients and A1 OC samples.

### 2.6. ML-Ddx4^+^ Cells Induce the Expression of Stemness Markers in A2780 OC Cells

**Figure 4:**
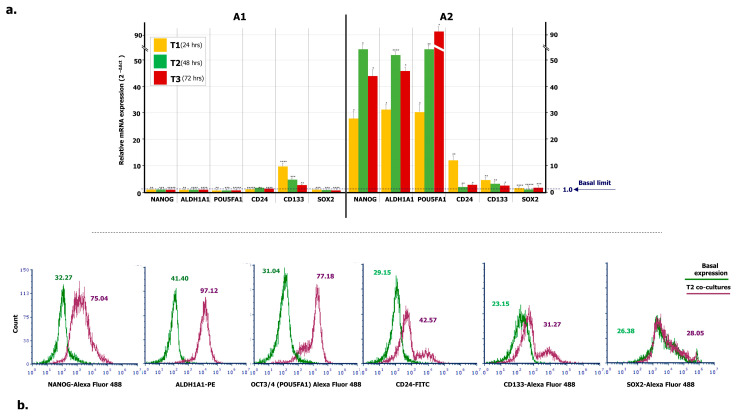
Expression of stemness markers in A2780 population conditioned by OC-derived ML-Ddx4^+^ cells. (**a**) RT-PCR was employed to measure mRNA levels of stemness genes in A2780 cell line incubated with ML-Ddx4^+^ cells from NS-EOC samples. Data were obtained using biological and technical triplicates and mRNA levels were normalized to GAPDH, used as an internal control. Gene expression was then evaluated in A2780 cells after 24, 48 and 72 h of co-culture and compared with relative basal levels by using the 2^-ΔΔct^ method. As shown, with the exception of CD133, after 24 h of co-culture with Ddx4^+^ cells from A1, the expression of the other stemness genes in A2780 remained similar to the baseline. By contrast, a general trend of increased expression levels was recorded at all time points when using ML-Ddx4^+^ cells from A2 OC samples. In particular, at T1 we observed increased mRNA levels of NANOG, ALDH1A1, POU5FA1, and CD24 with further variations at T2 and T3 (*p* < 0.01 in all instances), whereas both CD133 and SOX2 mRNAs maintained their basal expression. (**b**) Representative panels of flow cytometry hystograms depicting the differential protein expression of stem cell markers, evaluated on logarithmic *X*-axis as a shift of fluorescence peaks. Fluorescence intensities were revealed by Accuri C6 Plus proprietary analysis software and used as semi-quantitative parameter to evaluate variations in protein expression. The values related to protein expression were represented as MFI-R of the stained sample and its relative negative isotype control, and reported as MFI-R values. NANOG in co-culture (purple line) was expressed with higher MFI-R (75.04) with respect to the single culture (green line; MFI-R: 32.27). Moreover, ALDH1A1 and POU5FA1 showed a similar protein upregulation, whereas CD24 and CD133 were only slightly modulated. On the other hand, SOX2 expression was not further modified (Basal MFI-R = 26.38 vs. co-culture at T2, MFI-R = 28.05).

To verify the propensity of ML-Ddx4^+^ cells to trigger a stemness condition in A2780 tumor cells, we investigated the expression of several OC stem cell genes in A2780 cells, after co-culture with ML-Ddx4^+^ones from both OC patients’ groups, at different time points. Figure 4a summarizes the results. As shown, with the exception of slightly increased CD133 RNA levels in A2780 cells, after 24-h of co-culture with Ddx4^+^ cells from A1 OC samples, expression of the other stemness genes remained uniformly similar to their basal levels. By contrast, a general trend to increased gene expression levels was recorded at all time points when using the ML-Ddx4^+^ cells from A2 OC samples. In particular, at T1 we found a dramatic increment of mRNA expression of NANOG, ALDH1A1, POU5FA1, and CD24 with further variations at T2 and T3 (*p* < 0.01 in all instances), whereas both CD133 and SOX2 mRNAs maintained their basal expression levels.

Moreover, protein expression of the above-mentioned stem cell markers was evaluated by flow cytometry, at both basal levels and an intermediate time point of co-culture (T2), and reported as an MFI ratio (MFI-R) with respect to the relative isotype negative control. NANOG, ALDH1A1, POU5FA1, CD24 and CD133 protein expression was significantly increased in co-cultured A2780 cells (*p* < 0.05 in all instances), whereas the SOX2 molecule was apparently not modulated. Figure 4b shows a representative panel of flow cytometry hystograms depicting the differential protein expression, evaluated on the logarithmic *X*-axis as a shift of fluorescence peaks. As can be seen from the relative values of MFI-R, NANOG, ALDH1A1, POU5F1, CD24 and CD133 were variably upregulated, whereas the expression of SOX2 in co-cultures overlapped the basal levels. Even if based on a small number of co-cultures, these findings suggest that, after the acquisition of an ML-phenotype, Ddx4^+^ cells from highly invasive OCs are capable to prime the expression of stemness markers in A2780 cells. This effect, however, was not detected when using the same cells from stage I-II NS-EOCs, obtained from A1 subgroup.

### 2.7. ML-Ddx4^+^ Cells Variably Induce Functional Genes in A2780 OC Cells

**Figure 5:**
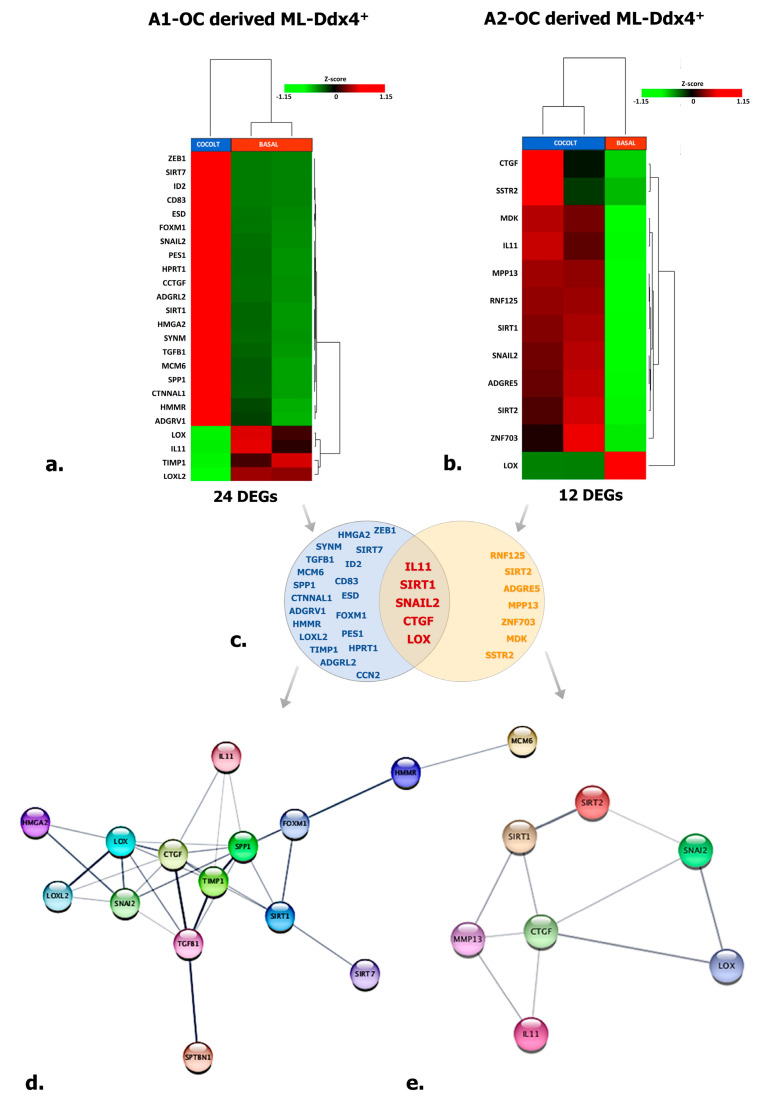
Targeted RNAseq of A2780 conditioned by ML-Ddx4^+^ cells. The image shows targeted RNAseq data depicting the expression of 118 genes enrolled in cancer cell processes in A2780 cells conditioned by ML-Ddx4^+^ cells from A1 (**a**) or A2 (**b**) NS-EOC. The heatmaps show both numbers and nomenclature of DEGs induced in A2780 by those cells (**a**,**b**), whereas the Venn diagram (**c**) depicts shared DEGs (−2 < FC < 2 (Fold Change); *p* ≤ 0.05 and False Discovery Rate (FDR) < 0.25) in A2780 OC cells differentially conditioned by ML-Ddx4^+^ cells from early-stage OC (A1) or advanced (A2) tumors. Functional gene networks of differentially expressed genes (DEGs) are represented as obtained by StringApp from Cytoscape (**d**,**e**). The results from this analysis support the hypothesis that, although at variable intensity, ML-Ddx4^+^cells from non-serous NS-EOCs are capable to deregulate a number of genes that are differentially enrolled in cancer biological processes.

To explore the effects of ML-Ddx4^+^cells on OC, we investigated by targeted RNAseq in next-generation sequencing (NGS) (see Materials and Methods) the expression profile of 118 functional genes in A2780 cells, after their co-culture with ML-Ddx4^+^ cells from either A1 or A2 OC patients’ groups. The analysis was performed at all co-culture time points (T1, T2, and T3), although significant variations were detected only after 48 h (T2). Tables 2–4 include lists of differentially expressed genes (DEGs) with relative fold-change values, induced by Ddx4^+^ cells. Figure 5a,b depict representative heatmaps of supervised hierarchical clustering of DEGs, emerged in A2780 cells after conditioning by ML-Ddx4^+^ populations, derived from either A1 (a) or A2 (b) OC patients. As shown, in both experimental conditions we observed a variable number of deregulated genes, mainly related to EMT, cellular adhesion, extracellular matrix remodeling, invasion and other cell activities, while a distinct cluster of genes was observed to be deregulated exclusively in cells co-cultured with ML-Ddx4^+^ cells from the A2 patient (Figure 5c). In particular, upregulated DEGs included *SIRT1*, which is involved in stemness [28] and chemoresistance [29], *SNAIL2,* which participates in EMT and collagen remodeling [30,31], and *SIRT2*, *CTGF*, *IL-11* and *MMP13*, involved in cell migration, invasiveness as well as resistance to platinum and paclitaxel [32,33,34,35,36]. Other upregulated DEGs, such as *MCM-6*, *TGFB-1*, *HMGA2* and *FOXM1*, are involved in similar cancer-related processes [37,38,39,40,41,42], while downregulated DEGs, such as *LOX* and *TIMP-1* (which are associated with poor cancer prognosis [43,44,45]), were observed in A2780 co-cultured with both A1 and A2 derived ML-Ddx4^+^cells. Among these gene expression alterations, only *ADGRL2* and *PES1* upregulation were observed in A2780 cells conditioned with control ML-Ddx4^+^ cells, in a similar fashion to what emerged from the A1-related co-culture.

**Figure 6:**
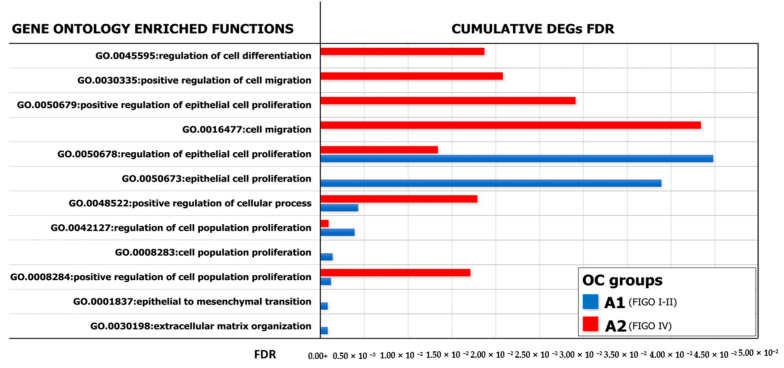
GO analysis of enriched biological processes. Representation of the Gene Ontology (GO) enriched functions (FDR < 0.05) revealed in A2780, conditioned by ML-Ddx4^+^ cells from either early- (blue) or advanced- (red) stage NS-EOC. The analysis showed differences in the extent of several biological processes, as induced by the two models of OC. In particular, functional enrichment analysis applied to DEGs identified that, in both patient groups, genes in the “biological process” GO category were significantly enriched in the terms “regulation of cell population proliferation”, “regulation of epithelial cell proliferation”, “extracellular matrix organization”, “cellular component organization” and “regulation of cell differentiation and cellular process”.

With the purpose of identifying a functional relationship among DEGs emerged from co-culture experiments, we have constructed specific networks, shown in Figure 5d,e, adopting StringApp on Cytoscape software (version 3.7.2) [46,47]. Pathway and functional enrichment analysis applied to DEGs identified that, for both patient groups, genes in the Gene Ontology (GO) category “biological process” were significantly enriched in the terms “regulation of cell population proliferation”, “regulation of epithelial cell proliferation”, “extracellular matrix organization”, “cellular component organization” and “regulation of cell differentiation and cellular process” (Supplementary Table S2). A graphical representation of these data is depicted in Figure 6 which shows that at least six out of twelve categories were enriched in A2780 cells co-cultured with ML-Ddx4^+^ cells from NS-EOCs, belonging to the A2 subgroup of patients. Several of these processes were virtually absent in cells stimulated with ML-Ddx4^+^ cells from the A1 patient, whereas others, such as “cell population proliferation”, “EMT” and “extracellular matrix organization”, remained apparently inactivated by this analysis.

These results provided evidence over the role of tumor-derived Ddx4^+^ cells in supporting cancer progression, at least in NS-EOCs, as suggested by the induction of transcriptome modifications.

## 3. Discussion

**Figure 7:**
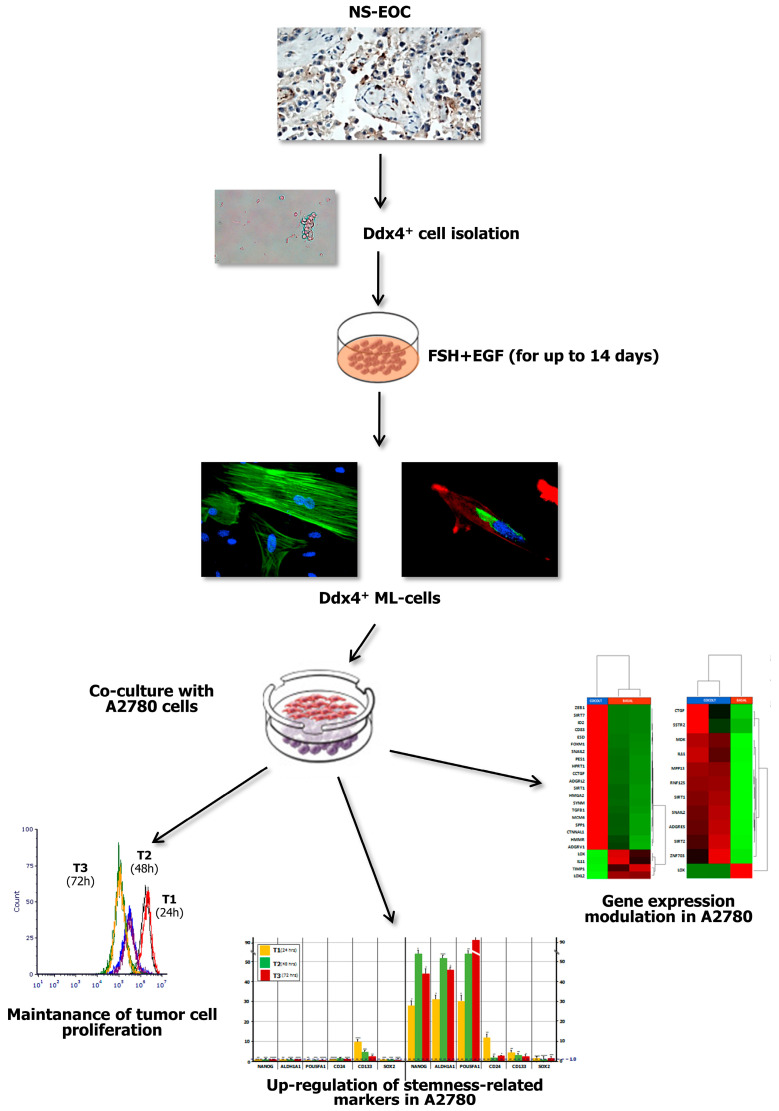
Summary of major experimental results. The present study investigated the role of Ddx4^+^ OSCs in post-menopausal women, focusing on the ovarian carcinogenesis and, in particular, on NS-EOC. Increased Ddx4 expression, evaluated by IHC, was demonstrated in samples from patients with advanced NS-EOC (A2), compared to those with early-stage disease (A1). Once the small round Ddx4^+^ cells were isolated from NS-EOC samples, we demonstrated that, in response to FSH + EGF stimulation, they differentiated to ML-cells, which exhibited spindle-like shape, stretched cytoskeleton and cytoplasmic Ddx4 localization. Co-culture of Ddx4^+^ ML-cells with A780 OC cells were thus performed, observing that the former maintained tumor cell proliferation, while inducing the acquisition of a “stem” and pro-metastatic phenotype by A2780 cells, as confirmed by transcriptomic analyses in targeted RNAseq.

Ddx4^+^ cells are regarded as OSCs and are also detectable in the post-menopausal ovarian cortex [11]. In animal models [48], as well as in human in vitro studies [11], these cells are postulated to support the neo-oogenesis, while showing high plasticity and capability to differentiate into somatic cells belonging to all three germ layers, under appropriate stimuli [4]. However, in relation to the reciprocal transdifferentiation with cells of mesenchymal lineage, Ddx4^+^ cells, as multipotent stem cells expressing major stemness markers [8], are usually considered ontogenetically incline to the mesodermal differentiation and efficient supporters of OC growth, since they are largely expressed by high-grade serous cancers [3]. Thus, while in our previous work we proved that Ddx4^+^cells from healthy post-menopausal women generate oocyte-like cells in vitro [11], data from the present study provide evidence that the same cells from highly aggressive NS-EOCs are activated as mesenchymal cells in response to FSH, and induce stemness genes in a model of NS OC, namely A2780 cells, while eliciting enriched gene functions typical of invasive OCs (Figure 7).

By investigating different cohorts of NS-EOCs in relation to their FIGO stage, similarly to serous cancers, we found that those from advanced clinical stages, namely FIGO IV, expressed the highest IHC levels of Ddx4. In fact, a mean value of 61.6 ± 12.5% was measured in OC samples from A2 patients, whereas only 17.4 ± 4.1% of those cells were detected in earlier OC stages of the A1 cohort. This result is probably enough to postulate that the Ddx4^+^ cell enrichment in OCs could be regarded as a high-grade tumor biomarker both in serous and non-serous tumors.

The somatic differentiation of Ddx4^+^ cells to ML-cells revealed by CD105, CD44, CD90, CD146, CD73 and VCAM1 expression has been reported as dependent on different stimuli [4]. In particular, FSH in association with valproic acid actively primes the in vitro growth of Ddx4^+^ cells with the formation of large cell clusters resembling tumor-like structures [14]. In our model, besides the intrinsic expression of mesenchymal gene mRNAs (Figure 2(a-iv)), in response to FSH we found a high propensity of Ddx4^+^ cells from NS-EOCs to over-express mesenchymal markers and acquire a fibroblast-like shape (Figure 2b). To this regard, the FSH sensitivity of Ddx4^+^ cells has been already reported [10], although the FSHR isoform promoting the ML patterns’ acquirement is presently undefined. Since the FSHR3 variant is apparently involved in a few biological processes, as premature ovarian failure and aging, as well as OC development [10], it is conceivable that, even in mesodermal differentiation, the FSHR3 isoform is expressed by Ddx4^+^ cells though this was not assessed in our study. However, with respect to other studies exploring the molecular aspects of stromal cells within the OC microenvironment, it seems that ML-Ddx4^+^ cells surrounding OC cells are different from the cancer associated mesenchymal stem cells (CA-MSCs) in relation to a separate gene expression profile of these cells [26], as well as to the diverse transcriptomic modifications induced by CA-MSCs or ML-Ddx4^+^ cells in OC cells [49]. On the other hand, the uniqueness of FSH-induced ML-Ddx4^+^ cell population is also sustained by the evidence that stromal cells from other derivations are variably responsive to FSH stimulation, despite the putative FSHR3 isoform expression [50,51].

The potential capability of MSCs to support tumor cell proliferation has been widely proved in most solid malignancies [26,27] including OC. In this regard, the cross-talk between mesenchymal and tumor cells has been shown to induce transcriptomic changes in the latter, characterized by the upregulation of genes involved in cell proliferation, migration and invasiveness that ultimately lead to the acquisition of a pro-metastatic phenotype [49]. On the other hand, Khalil and co-workers demonstrated that MSCs from different sources were capable to exert a pro-apoptotic effect in vitro on several OC lines, while decreasing tumor cell invasiveness and aggressiveness [52]. In our experimental model, by co-culturing A2780 with tumor-derived ML-Ddx4^+^ cells, we observed that the latter did not compromise OC cell viability, but preserved their proliferation rate, as confirmed by the transcriptomic variations that we observed in subsequent experimental steps.

Indeed, with the purpose of verifying whether or not ML-Ddx4^+^ cells were capable of inducing a stemness condition in A2780 cells, we investigated the RNA levels of stem-cell markers and found that ML-Ddx4^+^ cells from A2 OC samples significantly upregulated NANOG, ALDH1A1, CD24, CD133 and POU5FA1 in OC cells, since the first 24 h of co-culture (Figure 4). Similar data have also been obtained by using CA-MSCs that promoted in vitro the growth of several OC cell lines, such as SKOV3 or CAOV3 or PEO1, resulting in the formation of tumor spheres and development of chemoresistance, which are usually considered as peculiar features of cancer stemness [26]. Notably, ML-Ddx4^+^ cells from early stage OC (A1) were unable to induce similar effects on A2780 cells, thus suggesting that their stemness-priming potential is apparently variable in relation to the malignancy degree of the original cancer as a subsequent event of a theorized “positive feedback loop” [26].

Moreover, we also investigated the transcriptomic modifications of a panel of genes enrolled in major functions of the OC progression, and observed that ML-Ddx4^+^ cells from both OC subgroups, namely A1 and A2, deregulated in A2780 cells several key genes involved in ovarian carcinogenesis (Figure 5 and Table 2, Table 3 and Table 4). Among these, while in cells conditioned by ML-Ddx4^+^ cells from the A1 patient we revealed DEGs *FOXM1*, *HMGA2* and *TGF-B1* involved in the modulation of EMT, tumor cell migration and invasiveness [38,40,41], those related to the ML-Ddx4^+^ cells from the A2 patient included *ZFN703*, *SSTR2* and *MMP13*, which regulate OC proliferation, chemoresistance and prognosis [53,54,55]. However, besides these DEGs, which were separately registered in experiments using ML-Ddx4^+^ cells from A1 and A2 OC patients, several deregulated genes were shared between A2780 cells conditioned by each ML-Ddx4^+^population. Among these, upregulated genes included *SNAIL2*, enrolled in both EMT and collagen remodeling [30,31], *SIRT1*, which is an inducer of cancer stemness and chemoresistance [28,29], and *CTGF*, recently associated with poor prognosis in a few tumors, including OC [34]. We also observed an opposite deregulation of *IL-11*, since it was 3.56-fold down regulated in A2780 co-cultured with A1-derived ML-Ddx4^+^, as compared to a 3.44-fold up regulation with A2 ML-Ddx4^+^ cells. The role of this cytokine in the evolution of OC is presently unclear [56] though the alternate deregulation in our OC model, using ML-Ddx4^+^ cells from OCs at different stages, may theoretically suggest a negative prognostic significance of *IL-11* upregulation (Table 2). This possibility is also sustained by data obtained from control ML-Ddx4^+^ cells, which exhibited a very poor capability to reprogram the A2780 transcriptome.

With the purpose of verifying the functional gene networks induced in both experimental models of A2780 cells conditioned by ML-Ddx4^+^ cells from A1 and A2 OCs, we also investigated the GO of DEGs and found different enriched biological processes between both systems. As shown in Figure 6, the gene networks activated by ML-Ddx4^+^ cells from A2 OC were related to several functions, such as “cell proliferation”, “migration” and “control of cellular process”, which were defectively recurrent or virtually absent in A2780 cells after conditioning with the A1 OC derived ML-Ddx4^+^ cells.

Data from our work emphasize the possible role of Ddx4^+^ cells in OC progression. We have provided evidence that, once stimulated in vitro by FSH, these cells morphologically and functionally acquire an ML phenotype capable of modifying the transcriptome of OC cells in an apparently worsening evolution. Although experienced in vitro, our experimental model resembles several pathological conditions related to the post-menopausal age for the persistently increased levels of FSH, and the higher incidence of OCs during this time for the women. We have shown that ML-Ddx4^+^ cells from advanced non-serous malignancies are able to prime a stemness condition in cancer cells, that may at least partly explain their chemoresistance which leads in the clinical practice to poor prognosis in the late stage of the disease [57,58]. Even if produced in small cohorts of patients, data from our study deserve further investigation to explore the potential prognostic meaning of Ddx4^+^ cells in epithelial OCs. Based on our observation, at least the levels of Ddx4^+^ cells in OCs could be considered a putative novel biomarker of prognosis in these malignancies.

## 4. Materials and Methods

### 4.1. Patients and OC Cell Culture

Twenty-two post-menopausal women ranging 44–87 yrs of age and undergoing either laparoscopic or laparotomic surgery for benign gynecological diseases formed the control group, whereas the A group included 23 post-menopausal patients with NS-EOC. Thus, in relation to their clinical conditions, 9 patients with FIGO I-II stage OC were assigned to the A1 subgroup, while 14 patients showing the disease at FIGO IV stage converged in A2.

The OC patients from both A1 and A2 subgroups were enrolled at diagnosis and prior of any neoadjuvant chemotherapy or other systemic anti-cancer treatments. All patients were recruited during the last two years at the University-Hospital “Policlinico” of Bari (Italy) or at the National Cancer Institute (NCI) “Giovanni Paolo II-IRCCS” of Bari (Italy). All subjects gave their informed consent for inclusion before they participated in the study. The study was conducted in accordance with the Declaration of Helsinki, and the protocol was approved by the Ethics Committee of the above-mentioned Hospital (prot. # 5329; date of approval: 24 November 2017).

We also utilized A2780 OC cell line (Merck Life Science, Milan, Italy) after confirming the absence of Mycoplasma contamination (MP0040; Sigma-Aldrich, Milan, Italy). Cells were cultured in complete RPMI 1640 medium (10% fetal bovine serum plus 1% penicillin-streptomicin-glutamine; Gibco^®^, Waltham, MA, USA) and grown at 37 °C in a 5% CO_2_ incubator.

### 4.2. Ddx4 Detection in A2780 Cells and in OC Specimens

Ddx4 expression was primarily assessed in A2780 cells by flow cytometry. Briefly, cells were fixed and permeabilized by an Inside Stain kit (Miltenyi Biotec, Bergisch Gladbach, Germany) and 1 × 10^5^ viable cells were incubated with 5 μL of the primary antibody at 4 °C for 30 min, then a fluorescein isothyocianate (FITC)-conjugated anti-rabbit secondary antibody (Sigma-Aldrich, Milan, Italy) was added at 1:100 dilution. The isotype control was used as a negative parameter. The cell suspensions were then analyzed by Accuri C6Plus (Becton Dickinson, Milan, Italy) flow cytometer, using the proprietary software and FCS Express software (De Novo Software, Pasadena, CA, USA).

OC samples were analyzed by IHC to investigate Ddx4 expression. To this, formalin fixed paraffin embedded (FFPE) tumor sections were processed by an automated instrument (Bench Mark, ULTRA; Hoffmann-La Roche, Basel, CH Switzerland). Slides were de-waxed with the mild detergent EZ-Prep (Ventana Medical Systems, Hoffmann-La Roche) and pretreated with a combination of heat- and proteolytic-induced epitope retrieval steps. Sections were then incubated with an anti-Ddx4 primary antibody (Abcam ab13840, Cambridge, MA, USA) diluted 1:200, followed by biotinylated and peroxidase-labeled streptavidin secondary reagents (Vector Laboratories, Burlingame, CA, USA) and subsequent staining with chromogen substrate 3-3’-diaminobenzidine (DAB, Vector Laboratories). After counterstaining with Mayer’s hematoxylin, the slices were inspected under a Leica DM2500 microscope (Leica Microsystems, Milan, Italy), and Ddx4 expression was measured by a semi-quantitative assessment, considering the mean percentage of Ddx4^+^ cells in five random fields for each slide. In addition, the staining intensity was defined as + (weak), ++ (moderate) and +++ (strong), applying a three-tier ordinal categorical system.

### 4.3. Isolation of Ddx4^+^ Cells from OC Samples

OC pieces were digested by 1 mg/mL collagenase and 1 μg/mL DNAse I (Sigma-Aldrich) for 120 min at 37 °C. Cell pellets were then suspended in running buffer (Miltenyi Biotec) and incubated with 10 µL of rabbit anti-human Ddx4 antibody for 30 min at 4 °C. Thus, the samples were treated with anti-rabbit IgG microbeads (Miltenyi Biotec), and Ddx4^+^cells were isolated by an automated magnetic-activated cell sorting system (autoMACS Pro, Miltenyi Biotec,) as previously described [11]. The purity of cell population was assessed by the flow cytometry detection of membrane Ddx4. Briefly, an aliquot of isolated cells was incubated with rabbit anti-human Ddx4 antibody (Abcam ab13840) and then processed with an FITC-conjugated anti-rabbit antibody (Sigma-Aldrich). In addition, cytoplasmic expression of Ddx4 was also evaluated by flow cytometry, after cell fixation and permeabilization as described for A2780 cells. In each case, an isotype control was used as negative parameter.

### 4.4. Cultures of Ddx4^+^Cells and Subsequent Morphological and Phenotype Characterization

Ddx4^+^ cells were cultured for 14 days in the presence of 100 ng/mL FSH (Sigma-Aldrich) and 10 ng/mL EGF (Thermo Fisher, Waltham, MA, USA), in Dulbecco’s Modified Eagle Medium/Nutrient Mixture F-12 (DMEM-F12) (Thermo Fisher) containing 10% fetal bovine serum, 1% pen/strep-glutamine. Culture medium was replaced every 48–72 h, and the cell morphology was inspected daily by light microscopy, whereas potential phenotype variations, in relation to both FSH and EGF stimuli, as well as persistence of intracellular Ddx4 expression, were assessed by flow cytometry. To this, the cells were incubated with FITC-conjugated anti-EPCAM and anti-E-cadherin (eBioscience, Thermo Fisher) antibodies to detect the epithelial phenotype, whereas phycoerythrin (PE)-conjugated anti-CD146 (BD Pharmingen, San Diego, CA, USA) and anti N-Cadherin (eBioscience, Thermo Fisher) antibodies were employed for mesenchymal markers. In addition, cultured cells were stained with PE-conjugated anti-CD105, FITC-conjugated anti-CD73 and allophycocyanin (APC)-conjugated anti-CD90 antibodies (eBioscience, Thermo Fisher) as markers of MSCs. After their incubation, cell preparations were analyzed by Accuri C6Plus flow cytometer and dedicated softwares. Isotype controls were used as negative parameters.

We also investigated Ddx4 localization by inspecting the cultured cells under confocal inverted microscope, after appropriate staining. Thus, following their fixation and permeabilization, the cells were incubated with the above mentioned primary anti-Ddx4 antibody overnight at 4 °C and subsequently treated with an FITC-conjugated anti-rabbit secondary antibody (diluted 1:100) for one hour at room temperature. Finally, the cells were alternatively stained by tetramethylrhodamine (TRITC)- or FITC-conjugated phalloidin (Thermo Fisher) for actin visualization and by 4′,6-diamidino-2-phenylindole (DAPI, Sigma-Aldrich, Milan, Italy) for nuclei detection. The slides were analyzed by using Eclipse Ti-E confocal microscope and Nikon Instruments (NIS) element software (C2 plus; Nikon Instr., Lewisville, TX, USA).

To assess their postulated mesenchymal commitment, both Ddx4^+^ and negative cells from OC samples were investigated by quantitative PCR using multiplex TaqMan assays in a ddPCR system (QX200; Bio-Rad Laboratories). In particular, CD73-FAM (dHsaCPE5058548), CD90-FAM (dHsaCPE5029974) and CD105-HEX (dHsaCPE5049501) probes were used as markers of human MSCs, whereas GAPDH (qHsaCEP0041396) served as a housekeeping gene. Data were analyzed by QuantaSoft analysis software (Bio-Rad Laboratories, Hercules, CA, USA)).

### 4.5. Co-Culture of ML Ddx4^+^ Cells with A2780 Cells

To investigate the effect of ML-Ddx4^+^ cells, derived from either controls or OC patients, on A2780 growth we used a CFSE assay (Thermo Fisher). Briefly, A2780 cells were washed in PBS and stained with CFSE working dye solution, as described [59]. Labeled cells were centrifuged, resuspended in culture medium (DMEM-F12 containing 10% fetal bovine serum and 1% pen/strep-glutamine) and then seeded at 30,000 cells/well density in six-well plates, separated from ML-Ddx4^+^ cells (in 1:1 ratio) through 1.0 µm pored inserts (Becton Dickinson, NJ, USA). Co-cultures were performed in triplicates and grown for up to 72 h. At each time point (T1: 24 h; T2: 48 h; T3: 72 h), pored inserts were removed and A2780 cells were recovered for CFSE analysis. The CFSE signal was calculated in living cells as MFI using an Accuri C6Plus flow cytometer, and the proliferative stimulus on co-cultured tumor cells, compared to CFSE-stained A2780 cells at each time-point, was calculated as MFI variation.

### 4.6. In Search of Cancer Stem Cell Markers in Conditioned A2780 Cells

To verify whether or not ML-Ddx4^+^ cells were capable to prime a stemness condition in A2780 tumor cells [26], we investigated by qPCR the expression of major markers described in OC stem cells, namely *CD133*, *POU5FA1 (OCT3/4)*, *CD24*, *SOX2*, *NANOG* and *ALDH1A1*. To this, 100 ng of RNA from A2780 cells, both at baseline and after co-culture with ML-Ddx4^+^ cells obtained from three representative patients of each A1 and A2 subgroups, was reversely transcribed by Super Script^TM^ IV VILO^TM^ Master Mix (Life Technologies^TM^, Carlsbad, CA, USA). The cDNA was then amplified by Plus Real-Time PCR (Life Technologies Inc., Carlsbad, CA, USA) using specific TaqMan^®^ Assay for encoding the mentioned stemness genes in A2780 OC cells. The data were obtained using biological and technical triplicates, and mRNA levels were normalized to the *GAPDH* gene, used as an internal control. Gene expression was then evaluated in A2780 cells after 24, 48 and 72 h of co-culture and compared with relative basal levels by using the 2^−ΔΔct^ method.

Moreover, protein expression of the same stem cell markers was assessed by flow cytometry. Briefly, 1 × 10^5^ A2780 cells, in single culture or co-cultured with ML-Ddx4^+^ ones, were incubated with 5 µL of Alexa Fluor 488-conjugated anti-NANOG, anti-POUF5A1 (OCT3/4), anti-CD133 and anti-SOX2 (Thermo Fisher) antibodies, as well as with PE-conjugated anti-ALDH1A1 (Abcam) and FITC-conjugated anti-CD24 (Thermo Fisher). The samples were analyzed using an Accuri C6Plus flow cytometer using dedicated software and FCS Express Software to detect fluorescence intensities as a semi-quantitative parameter to evaluate variations in protein expression. Thus, values related to protein expression were represented as MFI-R between the stained sample and relative negative isotype controls. The analysis was assessed on experiments performed in triplicate.

### 4.7. Transcriptome Analysis of A2780 Cells Co-Cultured with ML-Ddx4^+^ Cells

In order to investigate the conditioning effect of ML-Ddx4^+^ cells on A2780 cells, a targeted RNAseq approach was developed to quantify the expression of 118 genes, selected for their involvement in cancer-related pathways and functions as EMT (n. 43), angiogenesis (n. 7), cell adhesion (n. 12), cytoskeleton/cell motility (n. 9), extracellular matrix remodeling (n. 17), cell–cell signaling and cytokine/chemokine interaction (n. 5), signal transduction-Wnt/β-catenin pathway (n. 8), immune response and escape (n. 11), and kinases (n. 6).

The primer-pool library was designed on the Ion Ampliseq Designer platform (https://www.ampliseq.com/browse.action) after identifying the genes of interest using Web tools as UNIPROT (http://www.uniprot.org/), GENECARD (http://www.genecards.org/), OMIM (http://omim.org/) and iPATH2 (pathway.embl.de/iPath2.cgi#). To this, the total RNA from 3 × 10^4^ A2780 cells, co-cultured with ML-Ddx4^+^ cells obtained from single patients of A1 and A2 groups, as well as from controls, was isolated by an RNeasy Mini Kit (Qiagen, Hilden, Germany) and quantified by the Qubit^®^ 3.0 fluorometer (Life Technologies™ Carlsbad, CA, USA).

Sequencing libraries were constructed using the Ion AmpliSeq™ Library Kit 2.0 as indicated in the Ion AmpliSeq™ RNA Library preparation user guide (Ion AmpliSeq™ Library Preparation, Quick Reference, Publication Number MAN0006735 Revision F.0) starting from 100 ng of total RNA. Both the quality and quantity of libraries, purified with Agentcourt AMPure XP (Beckman Coulter, Indianapolis, IN, USA) and assessed by an Ion Library TaqMan Quantitation Kit (Life Technologies) on the StepOne Plus system (Applied Biosystem, Foster City, CA, USA), were finally templated through both the Ion OneTouch™ 2 System and Ion OneTouch™ ES and were then sequenced on the NGS Ion Torrent PGM™ using Ion Torrent™ 318 chips.

Preliminary analysis of data from all samples was completed using the AmpliSeq RNA plugin available for Ion Torrent platforms. However, we performed RNAseq data analysis by using PartekFlow, a start-to-finish software analysis for NGS data, using analysis initiation with FASTQ files as each sequenced RNA sample containing the information of sequenced reads and the quality score for each nucleotide. Hence, high-quality reads were aligned to reference human genome version hg19 by using STAR software, and DEGs were revealed by the gene set analysis (GSA) method.

Each gene was associated with a relative FC whose statistical significance was expressed in terms of *p*-value (*p* ≤ 0.05). Ranked DEGs were then functionally assessed by GO analysis, as a bioinformatics tool that clusters genes in working classes such as ”biological process”, “molecular function” and ”cellular component” [60,61], in parallel with the Kyoto Encyclopedia of Genes and Genomes (KEGG) system for pathway analysis [62]. As a cut-off criterion, a *p*-value < 0.05 was defined. Finally, Cytoscape (version 3.7.2), a software platform for bioinformatics analysis [46], and its application tool StringApp [47] were used for visualizing the network and performing enrichment analysis.

The statistically significant DEGs were grouped in a hierarchical manner using the correlation distance and displayed by heatmaps. All correlation analyses were performed by Pearson coefficient and adjustment for multivariate analysis was completed by the Benjamini and Hochberg method (False discovery rate; FDR < 0.25). All DEG analyses were performed both at intra-group and inter-group levels, according to the different time points of RNA isolation. Moreover, other differences among patient groups, e.g., the mean number of isolated Ddx4^+^ cells/sample and the expression of mesenchymal and stemness genes, were assessed by Student’s t-test using *R* software (version 3.6.1), and considering a *p*-value < 0.05 as significance limit. For experiments performed in triplicate, data are presented as means ± standard deviation. 

## Figures and Tables

**Table 1:** Groups of patients, histotypes and mean values of intraovarian Ddx4^+^ expression.

Populations	Patients and Histotypes	Age (Median)	FIGO Stage (2014)	Ddx4^+^ Cells Detected by Immunohistochemistry (IHC)	
				% of Positive Cells	IHC Score
Controls Total: 22 pts	Uterine fibromatosis: 8 pts	53.5	Not applicable	Mean value: 1.61 ± 0.7%	+: 18 pts (81.8%) ++: 4 pts (18.2%) +++: 0 pts
	Urogenital prolapse: 11 pts	65			
	Follicular ovarian cyst: 3 pts	54			
A1-OC patients (FIGO I-II) Total: 9 pts	Clear cell carcinoma: 5 pts	61	Ic1-Ic3	Mean value: 17.4 ± 4.1%	+: 3 pts (33.3%) ++: 5 pts (55.5%) +++: 1 pts (11.1%)
	Mucinous carcinoma: 2 pts	53.5	Ic3-IIa		
	Endometroid carcinoma: 2 pts	51.5	IIb		
A2-OC patients (FIGO IV) Total: 14 pts	Clear cell carcinoma: 6 pts	68.5	IVb	Mean value: 61.6 ± 12.5%	+: 1 pts (7.1%) ++: 5 pts (35.7%) +++: 8 pts (57.1%)
	Mucinous carcinoma: 3 pts	74	IVa		
	Endometroid carcinoma: 5 pts	51	IVa		

**Table 2:** DEGs shared in A2780 cells after 48 h co-culture with ML-Ddx4^+^ cells from both patient groups.

	A1–Derived ML-Ddx4^+^ Cells			A2–Derived ML-Ddx4^+^ Cells		
Gene Symbol	*p*-Value	False Discovery Rate (FDR)	Fold Change(FC)	*p*-Value	False Discovery Rate (FDR)	Fold Change (FC)
SNAIL2	8.03 × 10^−5^	3.37 × 10^−4^	4.68	4.13 × 10^−8^	7.02 × 10^−7^	2.52
SIRT1	8.21 × 10^−4^	2.72 × 10^−3^	4.49	8.32 × 10^−9^	2.83 × 10^−7^	4.12
CTGF	6.44 × 10^−3^	1.93 × 10^−2^	3.68	1.89 × 10^−8^	4.28 × 10^−7^	4.10
IL11	1.45 × 10^−2^	3.98 × 10^−2^	−3.56	1.86 × 10^−7^	2.31 × 10^−6^	3.44
LOX	4.14 × 10^−15^	1.30 × 10^−13^	−50.06	3.44 × 10^−23^	2.34 × 10^−21^	−23.48

**Table 3:** DEGs emerged in A2780 cells after 48 h co-culture with A1-derived ML-Ddx4^+^ cells.

Gene Symbol	*p*-Value	FDR	FC
ID2	1.25 × 10^−11^	2.62 × 10^−10^	35.35
ESD	1.36 × 10^−7^	1.22 × 10^−6^	27.39
ADGRL2	1.84 × 10^−8^	2.32 × 10^−7^	12.58
HPRT1	5.96 × 10^−8^	6.26 × 10^−7^	11.68
FOXM1	5.82 × 10^−7^	4.07 × 10^−6^	9.20
PES1	1.22 × 10^−8^	1.92 × 10^−7^	7.27
CTNNAL1	6.92 × 10^−6^	3.96 × 10^−5^	5.72
SIRT7	2.71 × 10^−4^	1.01 × 10^−3^	5.40
SYNM	1.54 × 10^−4^	6.07 × 10^−4^	5.37
HMGA2	3.79 × 10^−6^	2.39 × 10^−5^	3.83
TGFB1	1.29 × 10^−5^	6.23 × 10^−5^	3.72
HMMR	7.64 × 10^−4^	2.68 × 10^−3^	3.32
SPP1	8.39 × 10^−3^	2.40 × 10^−2^	3.18
CD83	4.03 × 10^−2^	9.76 × 10^−2^	3.11
MCM6	3.45 × 10^−7^	2.72 × 10^−6^	2.98
ZEB1	2.66 × 10^−2^	6.70 × 10^−2^	2.41
ADGRV1	4.28 × 10^−2^	9.98 × 10^−2^	2.16
TIMP1	1.51 × 10^−29^	9.51 × 10^−28^	−2.01
LOXL2	1.74 × 10^−5^	7.82 × 10^−5^	−2.24

**Table 4:** DEGs emerged in A2780 cells after 48 h co-culture with A2-derived ML-Ddx4^+^ cells.

Gene Symbol	*p*-Value	FDR	FC
RNF125	6.56 × 10^−6^	6.37 × 10^−5^	2.85
ZNF703	1.16 × 10^−4^	8.75 × 10^−4^	2.72
SSTR2	3.81 × 10^−2^	9.34 × 10^−2^	2.58
SIRT2	8.66 × 10^−5^	7.36 × 10^−4^	2.55
MMP13	3.68 × 10^−2^	9.34 × 10^−2^	2.44
MDK	7.74 × 10^−4^	5.26 × 10^−3^	2.10
ADGRE5	9.00 × 10^−4^	5.57 × 10^−3^	2.10

## Supplementary Materials

The following are available online at https://www.mdpi.com/1422-0067/21/17/6096/s1, Table S1: Clinical details of OC patients. Table S2: GO enrichment analysis of differentially expressed genes in A2780 cells co-cultured with Ddx4+ cells from both patients’ subgroups.
