# Supplementary material for: DEAD-Box Helicase 4 (Ddx4)+ Stem Cells Sustain Tumor Progression in Non-Serous Ovarian Cancers
Source: Int J Mol Sci. 2020 Aug 24;21(17):6096. doi: 10.3390/ijms21176096 (PMC7503840; doi:10.3390/ijms21176096)
Supplement: Supplementary file 1 [file ijms-21-06096-s001.pdf]

GROUP	PATIENT ID	HISTOTYPES	AGE	FIGO 2014 STAGE	TUMOR EXTENSION
A1 subgroup	#1	Clear cell ovarian carcinoma	62	Ic1	Limited to one ovary and surgical spill
	#2	Clear cell ovarian carcinoma	63	Ic2	Limited to one ovary and on surface
	#3	Clear cell ovarian carcinoma	61	Ic1	Limited to one ovary and on surface
	#4	Clear cell ovarian carcinoma	58	Ic3	Limited to both ovaries and positive peritoneal washing
	#5	Clear cell ovarian carcinoma	56	Ic3	Limited to both ovaries and positive peritoneal washing
	#6	Mucinous ovarian carcinoma	55	Ic3	Limited to one ovary and positive peritoneal washing
	#7	Mucinous ovarian carcinoma	52	IIa	Extension on fallopian tubes
	#8	Endometrioid ovarian carcinoma	44	IIb	Extension on pelvic intraperitoneal tissue
	#9	Endometrioid ovarian carcinoma	59	IIb	Extension on pelvic intraperitoneal tissue
A2 subgroup	#10	Clear cell ovarian carcinoma	76	IVb	Hepatic and inguinal lymphnode metastases
	#11	Clear cell ovarian carcinoma	68	IVb	Hepatic and inguinal lymphnode metastases
	#12	Clear cell ovarian carcinoma	62	IVb	Hepatic and inguinal lymphnode metastases
	#13	Clear cell ovarian carcinoma	71	IVb	Splenic, inguinal, periaortic lymphnode metastases
	#14	Clear cell ovarian carcinoma	69	IVb	Splenic, inguinal, periaortic lymphnode metastases
	#15	Clear cell ovarian carcinoma	64	IVb	Hepatic, splenic, inguinal and periaortic lymphnode metastases
	#16	Mucinous ovarian carcinoma	87	IVa	Pleural effusion with positive cytology
	#17	Mucinous ovarian carcinoma	74	IVa	Pleural effusion with positive cytology
	#18	Mucinous ovarian carcinoma	63	IVa	Pleural effusion with positive cytology
	#19	Endometrioid ovarian carcinoma	48	IVa	Pleural effusion with positive cytology
	#20	Endometrioid ovarian carcinoma	53	IVa	Pleural effusion with positive cytology
	#21	Endometrioid ovarian carcinoma	51	IVa	Pleural effusion with positive cytology
	#22	Endometrioid ovarian carcinoma	46	IVa	Pleural effusion with positive cytology
	#23	Endometrioid ovarian carcinoma	68	IVa	Pleural effusion with positive cytology

Supplementary Table S1 - Clinical details of OC patients.

GO enriched functions related to DEGs induced by ML-Ddx4^{+} cells from subgroup A1 of OCs

GO Term	Description	FDR	Genes
GO.0030198	Extracellular matrix organization	8.40E-04	*TIMP1,TGFB1,LOX,CTGF,LOXL2,SPP1*
GO.0001837	epithelial to mesenchymal transition	8.40E-04	*TGFB1,LOXL2,SNAIL2,HMGA2*
GO.0008284	positive regulation of cell population proliferation	0.0012	*SIRT1,TIMP1,TGFB1,ID2,IL11,FOXM1,CTGF,HMGA2*
GO.0008283	Cell population proliferation	0.0014	*TGFB1,ID2,HPRT1,PES1,CTGF,LOXL2,HMGA2*
GO.0042127	regulation of cell population proliferation	0.0039	*SIRT1,TIMP1,TGFB1,ID2,IL11,FOXM1,CTGF,SNAIL2,HMGA2*
GO.0048522	positive regulation of cellular process	0.0043	*SIRT1,TIMP1,TGFB1,ID2,IL11,HPRT1,SIRT7,FOXM1,SPTBN1,CTGF,CD83,LOXL2,SPP1,SNAIL2,HMGA2*
GO.0050673	epithelial cell proliferation	0.0389	*ID2,LOXL2*
GO.0050678	regulation of epithelial cell proliferation	0.0448	*SIRT1,TGFB1,SNAIL2*

GO enriched functions related to DEGs induced by ML-Ddx4^{+} cells from subgroup A2 of OCs

GO Term	Description	FDR	Genes
GO.0042127	regulation of cell population proliferation	9.3E-4	*SIRT1,SIRT2,IL11,ZNF703,SSTR2,CTGF,SNAIL2*
GO.0016477	cell migration	0.0434	*CTGF,SNAIL2,MDK*
GO.0050679	positive regulation of epithelial cell proliferation	0.0291	*SIRT1,ZNF703*
GO.0030335	positive regulation of cell migration	0.0208	*SIRT1,ZNF703,SNAIL2*
GO.0045595	regulation of cell differentiation	0.0187	*SIRT1,SIRT2,ZNF703,CTGF,SNAIL2*
GO.0048522	positive regulation of cellular process	0.0179	*SIRT1,RNF125,SIRT2,IL11,ZNF703,CTGF,SNAIL2,MDK*
GO.0008284	positive regulation of cell population proliferation	0.0171	*SIRT1,IL11,ZNF703,CTGF*
GO.0050678	regulation of epithelial cell proliferation	0.0134	*SIRT1,ZNF703,SNAIL2*

Supplementary Table S2 - GO enrichment analysis of differentially expressed genes in A2780 cells co-cultured with Ddx4^{+} cells from both patients' subgroups.
